# Determination of the Molecular Basis for a Limited Dimorphism, N417K, in the *Plasmodium vivax* Duffy-Binding Protein

**DOI:** 10.1371/journal.pone.0020192

**Published:** 2011-05-24

**Authors:** Amy M. McHenry, Samantha J. Barnes, Francis B. Ntumngia, Christopher L. King, John H. Adams

**Affiliations:** 1 Eck Institute for Global Health, University of Notre Dame, Notre Dame, Indiana, United States of America; 2 Department of Global Health, University of South Florida, Tampa, Florida, United States of America; 3 Department of Epidemiology and Biostatistics, University of South Florida, Tampa, Florida, United States of America; 4 Center for Global Health and Diseases, Case Western Reserve University, Cleveland, Ohio, United States of America; Walter & Eliza Hall Institute, Australia

## Abstract

Invasion of human red blood cells by *Plasmodium* merozoites is vital for replication and survival of the parasite and, as such, is an attractive target for therapeutic intervention. Merozoite invasion is mediated by specific interactions between parasite ligands and host erythrocyte receptors. The *P. vivax* Duffy-binding protein (PvDBP) is heavily dependent on the interaction with the human Duffy blood group antigen/receptor for chemokines (DARC) for invasion. Region II of PvDBP contains many allelic polymorphisms likely to have arisen by host immune selection. Successful vaccine development necessitates a deeper understanding of the role of these polymorphisms in both parasite function and evasion of host immunity. A 3D structure of the homologous *P. knowlesi* DBP predicts that most variant residues are surface-exposed, including N417K, which is a dimorphic residue change that has previously been shown to be part of a linked haplotype that alters DBP sensitivity to inhibitory antibody. In natural isolates only two residues are found at this site, asparagine (N) and lysine (K). Site-directed mutagenesis of residue 417 was used to create a panel of 20 amino acid variants that were then examined for their binding phenotype and response to immune sera. Our results suggest that the observed dimorphism likely arose due to both structural requirements and immune selection pressure. To our knowledge, this is the first exhaustive examination of this kind of the role of a single amino acid residue in antigenic character and binding ability. Our results demonstrate that a single amino acid substitution can dramatically alter both the ability of the PvDBP to bind to human erythrocytes and its antigenic character.

## Introduction

*Plasmodium vivax* is responsible for 70–80 million cases of clinical malaria annually and has a wide distribution that causes more than 50% of malaria cases outside of Africa [1]. Increasing reports of parasite drug resistance as well as cases of severe clinical disease due to *P. vivax* emphasize the need for better prevention and treatment strategies [2]–[5]. The Duffy binding protein (DBP) is a cysteine rich protein located in the micronemes of the *P. vivax* merozoites [6], [7]. It is believed to be released from the micronemes during initial attachment of the merozoite to the erythrocyte and is required for junction formation which is necessary to complete the invasion process [8]. DBP is an attractive vaccine target because of its nearly absolute requirement for invasion of host erythrocytes and because antibodies that recognize this molecule correlate with protection against infection [9], [10]. DBP contains the prototypical Duffy-binding ligand (DBL) domain or region II, which is a cysteine-rich region (12 consensus cysteines) responsible for receptor recognition in a wide variety of parasite cytoadhesion proteins [7]. A site critical for erythrocyte receptor (Duffy antigen/receptor for chemokines, or DARC) recognition has been mapped to an area between cysteines 4 and 7 of the DBL domain [11]–[13]. Interestingly, this is also the most highly polymorphic region of the entire open reading frame with a high ratio of nonsynonymous to synonymous polymorphisms, suggesting positive selection indicative of immune pressure [14]–[16]. In a similar manner, examination of the non-homologous proteins *Influenza* hemagglutinin (HA) and *Plasmodium* apical membrane antigen 1 (AMA-1) reveals a pattern of polymorphisms located adjacent to and surrounding their putative receptor binding sites. A consensus viewpoint interprets these substitutions as making it more difficult for host inhibitory antibodies, elicited by previous exposure to the pathogen, to recognize new variant epitopes and block the interaction between the pathogen ligand and the host receptor [17]–[22]. We hypothesize that the same mechanism of immune evasion operates to drive allelic diversity of DBP.

In previous studies we analyzed the variant alleles of field isolates from Papua New Guinea and determined that several polymorphisms (N417K, W437R, I503K) formed a linked haplotype [23]. This haplotype was shown to be important in determining the antigenic character and sensitivity of DBP to antibody inhibition. DBP containing 417K, 437R and 503K were refractory to inhibition with antiserum while DBP containing 417N, 437W and 503I were sensitive to inhibition. This result indicated that N417K forms part of an important haplotype that alters the antigenic character of DBP while other data supports that the N417K variation has special significance. Mapping of N417K onto a *P. vivax* DBP homology model based on the *P. knowlesi* DBPα crystal structure reveals that this residue is immediately adjacent to a motif identified by mutational analysis to be important for DARC receptor recognition [24], [25]. In addition, variation at residue 417 is limited to either N or K in all field isolates examined. Therefore, the objective of this research is to provide additional experimental rationale to account for the limited dimorphism at this residue. We hypothesize that functional requirements limit the type of substitutions at this site because other amino acids will interfere with the binding of the parasite ligand to the erythrocyte receptor. Alternatively, we hypothesize that positive immune pressure selects for compatible amino acids that also alter antigenic character in functionally important residues. Our results demonstrate that single amino acid substitutions at this site have a significant impact on the antigenic character of the PvDBP but variation is limited by functional constraints to bind the human erythrocyte receptor.

## Results

Our previous study of genetic analysis of *pvdbp* in clinical isolates has shown that variation at residue 417 is often linked to residues 437 and 503 [23]. We updated this analysis by examining 292 available PvDBPII sequences (Table 1). Residues 417 and 437 were linked 94.7% of the time, while residues 417 and 503 were linked 70.5% of the time. Analysis of this dataset indicated a total of 55 polymorphic sites with 50 dimorphisms, 4 trimorphisms and 1 site with four alternate residues.

**Table 1:** Association of polymorphisms N417K, W437R and I503K in 292 PvDBP isolates.

	Number of Isolates (% Frequency)	with 417K	with 437R	with 503K	with either
**417K**	132 (45.2%)^a^	—	125 (94.7%)^b^	93 (70.5%)^b^	127 (96.2%)^b^
**437R**	146 (50.0%)^a^	125 (85.6%)^c^	—	106 (72.6%)^c^	140 (95.9%)^c^
**503K**	145 (49.7%)^a^	93 (64.1%)^d^	106 (73.1%)^d^	—	108 (74.5%)^d^

a % frequency determined from total of 292 isolates.

b % frequency determined from number of 417K isolates.

c % frequency determined from number of 437R isolates.

d % frequency determined from number of 503K isolates.

**Figure 1:**
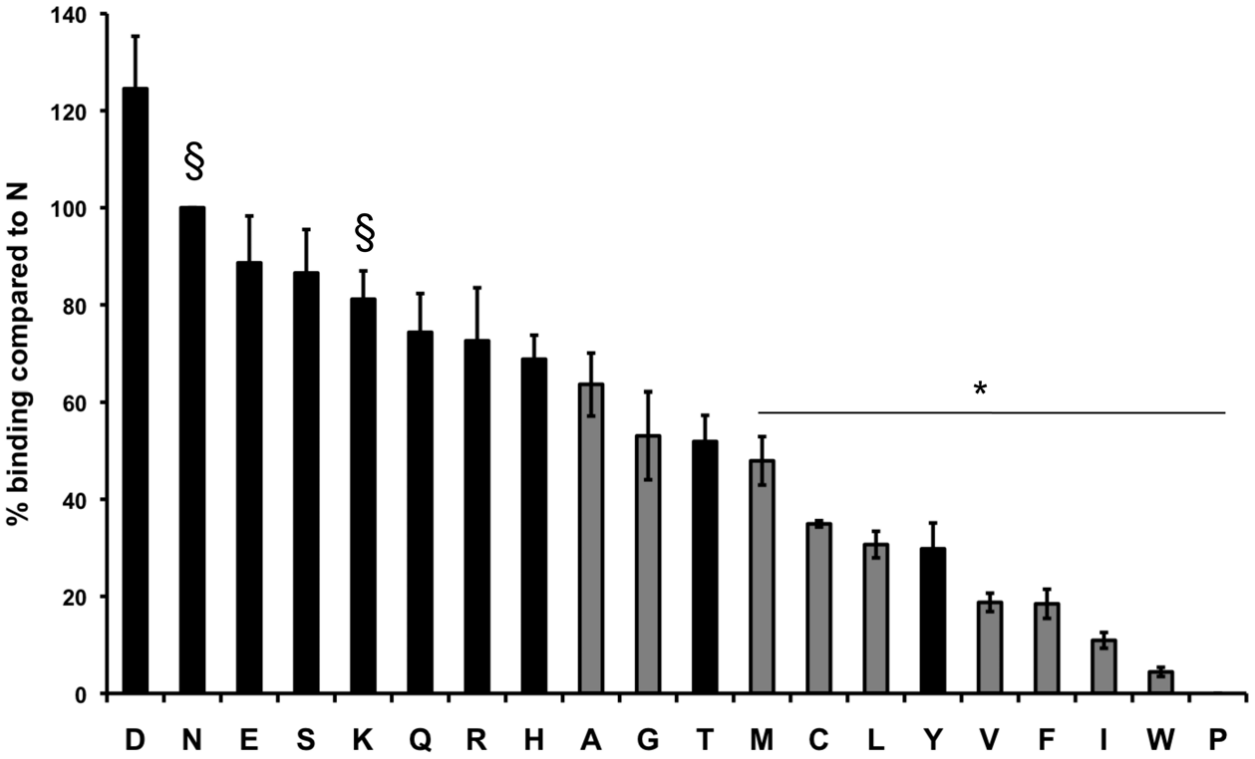
Percentage erythrocyte binding to DBP variants containing single amino acid substitutions at the 417 site. Binding is shown as a percentage compared to the naturally occurring variant N. All constructs are on a Sal I strain background. Polar residues are shown in black. Nonpolar residues are shown in gray. § indicates naturally occurring variant containing N or K at the 417 site. * indicates DBP variants which have significantly lower binding than one or both of the naturally occurring residues (N and K) (p<0.05). Statistical differences were analyzed using a 1-way analysis of variance (ANOVA) and a Tukey's posttest.

**Figure 2:**
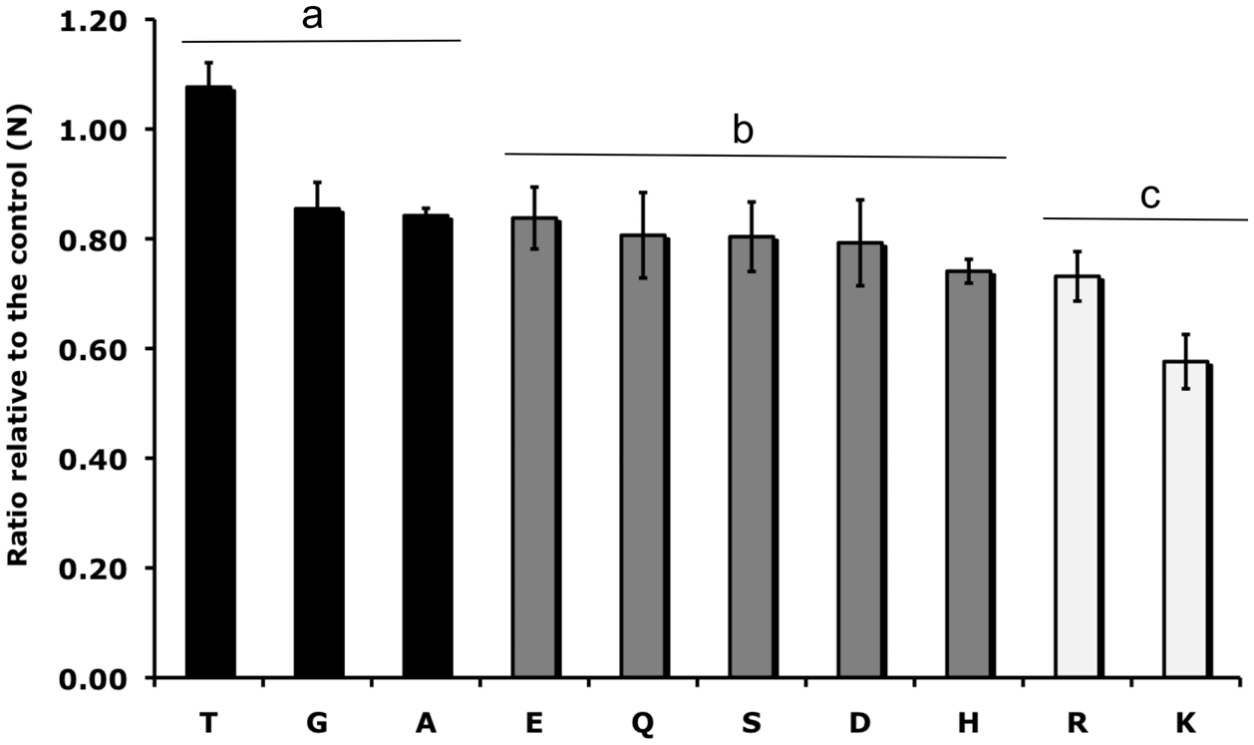
Inhibition of binding to DBP variants containing single amino acid substitutions at the 417 site. Sera raised against Sal I DBP (containing N417) was tested for its inhibitory efficacy against variant DBP forms with single amino acid changes created on a Sal I strain background using site-directed mutagenesis. Inhibition ratios were calculated by dividing the percentage inhibition against the variant DBP by the percentage inhibition against the Sal I control DBP. ^a^ DBP variants which were not significantly different from the Sal I (N) control (p>0.05). ^b^ DBP variants which were significantly different from both N and K (p<0.05). ^c^ DBP variants which were significantly different from N (p<0.05), but not significantly different from K (p>0.05). Statistical differences were calculated using a Dunn's multiple comparisons t-test with a Bonferroni correction for multiple comparisons.

We analyzed the binding phenotype of variant constructs containing each of the 20 amino acids at site 417 using an *in vitro* COS7 cell assay for DBP-erythrocyte binding function (Fig. 1). Nine of the constructs bound significantly less than the naturally occurring residues, N and K (p<0.05). Most of these residues were nonpolar although Y was also a poor binder. Nine of the variants, including two nonpolar residues (A and G), were not significantly lower than the naturally occurring residues in their binding phenotype (p>0.05) and did not bind to Duffy negative cells. We further analyzed these nine ‘normal binding’ variants for their inhibition phenotypes using polyclonal rabbit sera raised against the Sal I strain DBPII containing N at the 417 site (Fig. 2). This well characterized serum inhibits *in vitro* binding of DBPII to erythrocytes and the DARC receptor, as well as inhibits invasion of human erythrocytes by *P. vivax* parasites [26].

The nine ‘normal binding’ variant residues tested for sensitivity to anti-DBP antibody inhibition showed intermediate levels of inhibition between the naturally occurring residues, N and K, with the exception of T (Fig. 2). Three variants (T, G, and A) were not significantly different from naturally occurring variant N (p>0.05), but were significantly different from naturally occurring variant K (p<0.05). Five variants (E, Q, S, D and H) were significantly different from both N and K (p<0.05). Variant R was significantly different from N (p<0.05), but was not significantly different from naturally occurring residue K (p>0.05).

The presence of certain polymorphic residues in natural isolates may be affected by amino acid frequencies. Therefore, amino acid frequencies for the residues in the ‘normal binding’ variants were determined from a total of 421 *P. vivax* coding sequences (with a total of 295609 residues) (Table 2) [27]. Frequencies of these amino acids were also determined for the Sal I PvDBP coding sequence (with a total of 1070 residues) (Table 2). In the larger dataset, K, E, S, A and N were the most abundant residues. In the Sal I PvDBP data set K, N, S, D and E were the most abundant residues. Table 2 also includes the log odds score for substitution of each amino acid for N in a membrane protein [28]. Positive scores imply a biochemically favorable substitution, while negative scores imply a disfavored substitution. Variants are listed in decreasing order of their inhibition ratio (Fig. 2).

**Table 2:** Amino acid frequency in 421 *P. vivax* CDS and Sal I DBP CDS.

Amino Acid^a^	Frequency (%) in 421 *P. vivax* CDS^b^ [27]	Frequency (%) in the Sal I DBP CDS	% Binding relative to N	Log Odds Score for substitution for N^c^
T	5.27	6.36	52	1
***N***	***6.76***	***9.53***	***100***	***–***
G	5.64	6.64	53	−2
A	6.80	5.14	64	−1
E	8.86	7.66	89	1
Q	3.54	2.71	74	3
S	7.07	9.07	87	2
D	5.48	8.13	125	6
H	1.72	1.68	69	3
R	2.74	4.39	73	2
***K***	***10.51***	***9.91***	***81***	***5***

a Residues are shown in decreasing order of their inhibition ratios (See Fig. 2). Naturally occurring residues are in bold and italic.

b Codon usage information can be viewed at http://www.kazusa.or.jp/e/resources/database.html.

c Log odds scores are shown for substitution of N in a membrane protein [28].

A bias in codon usage for residue 417 may also affect amino acid substitutions. The codons for variant R that has a binding phenotype significantly different from naturally occurring residue N (p<0.05) but not from naturally occurring residue K (p>0.05), were analyzed (Table 3). Mutation of residue 417 from N to K requires a single base change. In contrast, mutation of residue 417 from N to R requires a double or triple base change of the amino acid codon.

**Table 3:** Analysis of codons required to alter N to K or R.

Mutation	Codons(p) indicates preferred codon	Number of nucleotides required for change
**N→K**	AAT→AAA AAC→AAG	1
**N→R**	AAT→AGA (p)AAC→AGG (p)AAT→CGT AAC→CGC AAT→CGA AAC→CGG	223

## Discussion

Vaccine development against malaria has achieved limited success so far. A number of challenges need to be overcome in designing a highly effective vaccine, especially the challenge of strain-specific immunity. Some antigens like the *P. vivax* DBP offer the potential of vulnerable epitopes associated with functionally sensitive motifs required for receptor recognition. However, the large number and diversity of polymorphisms found in the regions of the DBP necessary for ligand recognition and binding suggest that the parasite is very capable of adapting to evade any inhibitory host immune response [14]–[16], [29]–[31].

Consistent with this possibility is the previous work that demonstrated that strain-specific antibody inhibition can be mediated by polymorphic residues found in a linked haplotype [23]. We became interested in residue 417 because of its role in this strain-specific immunity and its importance in binding to DARC. We updated our previous linked haplotype data by examining 292 DBP sequences to determine the association of polymorphisms at the 417, 437 and 503 sites (Table 1). Although the linkage between these three residues is substantial, we chose to focus on the single amino acid change at residue 417 separately from the other polymorphisms for the following reasons. Firstly, through modeling based on the crystal structure of homologous *P. knowlesi* α DBL we found that residue 417 is located adjacent to a motif identified in two separate studies as important for recognition of the erythrocyte receptor [24], [25], [32]. Secondly, analysis of field isolates indicated that only amino acids N and K are found at residue 417 (Table 1). We hypothesized that multiple polymorphisms at this site must be limited by other factors, possibly including both functional constraints and/or the pressure of immune selection.

Binding analysis of variant constructs representative of all 20 amino acid possibilities indicates a functional constraint for nine of the non-naturally occurring residues (Fig. 1), indicating that a single amino acid substitution can drastically alter the ability of *Pv*DBPII to bind to the human erythrocyte. The poor erythrocyte binding properties of these amino acid substitutions would seem to be sufficient to preclude their occurrence on this critical parasite ligand.

We performed inhibition analysis on the remaining eleven constructs using antisera raised against the Sal I *P. vivax* strain containing N417 [26]. A number of residues were significantly different from N in their inhibition phenotype including E, Q, S, D, H, R and K. The two naturally occurring amino acids N and K have the most extreme antigenic differences (Fig. 2).

We calculated amino acid frequencies of the residues in the ‘normal binding’ variants to determine if their scarcity might suggest a possible explanation for their absence in natural isolates (Table 2). Although, amino acid frequency may explain the absence of a few of these polymorphic variants (for example, H or Q), it is not adequate to explain the absence of other residues such as E, S or D. Residues E, S and D are among the most frequently occurring residues in *P. vivax* CDS (Table 2), have positive log odds scores for substitution for N (Table 2), display binding phenotypes intermediate to or greater than the two naturally occurring residues (Fig. 1) and are significantly different antigenically from N and K (p<0.05) (Fig. 2) and yet they are absent in natural isolates.

One non-naturally occurring residue (R) was significantly different in its inhibition phenotype from N (p<0.05), but not from K (p>0.05). We examined this residue further to try to determine its absence from natural isolates. The much lower frequency at which this amino acid appears in *P. vivax* CDS may partially explain its absence in natural isolates (Table 2). In addition, analysis of the nucleotide codons that encode for this amino acid suggests a genetic explanation (Table 3). Although the change from N to R would result in a significant change in antigenicity, it would require a mutation of two or three nucleotide bases. The change from N to K, the two naturally occurring residues, results in the most extreme alteration of antigenic character and is also the simplest to achieve with a single nucleotide change.

In conclusion, these data demonstrate the impact a single amino acid substitution can have on both the ability of the PvDBPII to bind to human erythrocytes and the antigenic character of this vital invasion protein. In addition, these data provide a partial explanation for the absence of multiple polymorphisms at residue 417. A number of residues are eliminated from natural isolates because of functional constraints in their ability to bind to the red blood cell receptor. Other residues bind to erythrocytes in a similar fashion to the naturally occurring variants, but are not antigenically distinct from naturally occurring residue N, so there may be a lack of positive immune selection driving their appearance in natural isolates. Residue R, like naturally occurring residue K, has a more extreme difference in antigenic character as compared to residue N, but is a less abundant residue in *P. vivax* and is genetically more difficult to achieve spontaneously. Examination of the codons for these amino acids indicates that K is the simplest change to achieve with a single nucleotide substitution necessary to alter the codon. Other factors that may affect the absence of multiple polymorphisms at this site include the possibility that the partially linked residues at site 437 and 503 limit the appearance of certain residues at site 417. To our knowledge, this is the first exhaustive work of this kind to examine the impact of substitutions at a single residue on binding and antigenic character. These data provide experimental support for the hypothesis that positive immune selection pressure plays a role in the appearance of polymorphisms in functionally important residues of *P. vivax* DBP.

## Methods

### pEGFP-DBPII constructs & site-directed mutagenesis

Salvador I (Sal I) DBPII was cloned into the pEGFP-N1 plasmid with flanking signal sequences from the herpes simplex virus glycoprotein D1 allowing expression of a GFP fusion protein on the surface of transiently transfected COS7 cells (American Type Culture Collection, Manassas, VA). Mutagenesis to create a panel of N417 variants was performed using the Stratagene Quickchange mutagenesis kit (Stratagene, La Jolla, CA) as previously described [12], [23], [33], [34]. The pEGFP-DBPII plasmid containing DBPII cloned from the Sal I strain of *P. vivax* (containing N at the 417 location) was used as the parent template. Single residue changes were performed at the 417 site to create a panel of variants representative of all 20 amino acids all on the same genetic Sal I background. Recombinant plasmid DNA was purified using an endotoxin-free plasmid DNA purification system (Qiagen, Valencia, CA).

### COS7 cell binding & inhibition assays

COS7 (green monkey kidney epithelial) cells were maintained in Dulbecco's modified Eagle's medium (DMEM, Sigma, St. Louis, MO) containing 10% fetal bovine sera (FBS). Only cells between the passage numbers of 5 and 20 were used for binding and inhibition assays.

COS7 cells were plated in 24-well plates at a density of 35,000 cells per well and were transiently transfected with endotoxin-free pEGFP-DBPII DNA using Lipofectamine or Lipofectamine 2000 (Invitrogen, Carlsbad, CA) according to the manufacturer's instructions. Forty-two hours post-transfection, the transfected COS7 cells were incubated with DARC-positive human erythrocytes for 2 hours (2.5×10^7^ cells/well, previously washed three times with incomplete DMEM). Wells were washed three times with PBS to remove nonadherent erythrocytes and binding was scored by counting the number of rosettes per 30 fields of view at 200× magnification. GFP surface expression was observed to be consistent between all constructs suggesting comparable expression levels. Inhibition assays were carried out in the same manner except that transfected COS7 cells were preincubated for 1 hour at 37°C, 5% CO_2_ with antiserum diluted in incomplete DMEM prior to addition of the erythrocyte suspension. Antiserum against recombinant Sal I DBPII was produced as previously described [26]. Results from binding assays are shown as a percentage compared to the control (containing N at the 417 site). Percentage inhibition was calculated for each sample by comparing binding in the presence and absence of serum. A normalized inhibition ratio was calculated for each sample by dividing the percentage inhibition of the experimental sample by the percentage inhibition of the control sample. All binding experiments were carried out on at least two different clones for each sample and tested at least three times in triplicate. Inhibition experiments were carried out at least three times in triplicate.

### Analysis of PvDBPII sequences

All available PvDBPII sequences in the NCBI Protein Database were downloaded on 03/26/2010 and aligned using ClustalW. A total of 292 sequences were examined at residues 417, 437 and 503 to determine whether polymorphisms at these sites were linked.

### Modeling & statistical analysis

Modeling was done using the MacPymol Molecular Graphics System (DeLano Scientific LLC, San Carlos, CA, USA) and SWISS-MODEL (http://swissmodel.expasy.org/SWISS-MODEL.html) [35], [36]. Statistical analyses were performed using the Prism 4 program (GraphPad Software, La Jolla, CA) and the SAS 9.2 program (Cary, NC, USA released 2008). For binding assays, the results are shown as a percentage of the reference residue N. The percentages were analyzed using a 1-way analysis of variance (ANOVA) and a Tukey's posttest. Residues with binding phenotypes significantly lower than the two naturally occurring residues (N and K) (p-value≤0.05) were excluded from inhibition analysis. For inhibition assays, an inhibition ratio was determined by dividing the mean percentage of each residue by the mean percentage of the reference residue N to normalize the data. To determine whether variant residues were significantly different in their inhibition phenotypes from the natural occurring residues (N and K), a Dunn's multiple comparisons t-test was performed with a Bonferroni correction for multiple comparisons. Differences from the multiple comparisons test were found to be statistically significant at a p-value of 0.05 or less.

## References

[1] Mendis K, Sina BJ, Marchesini P, Carter R (2001). The neglected burden of Plasmodium vivax malaria.. Am J Trop Med Hyg.

[2] Baird JK (2004). Chloroquine resistance in Plasmodium vivax.. Antimicrob Agents Chemother.

[3] Ruebush TK, Zegarra J, Cairo J, Andersen EM, Green M (2003). Chloroquine-resistant Plasmodium vivax malaria in Peru.. Am J Trop Med Hyg.

[4] Kochar DK, Saxena V, Singh N, Kochar SK, Kumar SV (2005). Plasmodium vivax malaria.. Emerg Infect Dis.

[5] Tjitra E, Anstey NM, Sugiarto P, Warikar N, Kenangalem E (2008). Multidrug-resistant Plasmodium vivax associated with severe and fatal malaria: a prospective study in Papua, Indonesia.. PLoS Med.

[6] Adams JH, Hudson DE, Torii M, Ward GE, Wellems TE (1990). The Duffy receptor family of Plasmodium knowlesi is located within the micronemes of invasive malaria merozoites.. Cell.

[7] Adams JH, Sim BK, Dolan SA, Fang X, Kaslow DC (1992). A family of erythrocyte binding proteins of malaria parasites.. Proc Natl Acad Sci U S A.

[8] Miller LH, Mason SJ, Clyde DF, McGinniss MH (1976). The resistance factor to Plasmodium vivax in blacks. The Duffy-blood-group genotype, FyFy.. N Engl J Med.

[9] Wertheimer SP, Barnwell JW (1989). Plasmodium vivax interaction with the human Duffy blood group glycoprotein: identification of a parasite receptor-like protein.. Exp Parasitol.

[10] King CL, Michon P, Shakri AR, Marcotty A, Stanisic D (2008). Naturally acquired Duffy-binding protein-specific binding inhibitory antibodies confer protection from blood-stage Plasmodium vivax infection.. Proc Natl Acad Sci U S A.

[11] Ranjan A, Chitnis CE (1999). Mapping regions containing binding residues within functional domains of Plasmodium vivax and Plasmodium knowlesi erythrocyte-binding proteins.. Proc Natl Acad Sci U S A.

[12] VanBuskirk KM, Sevova E, Adams JH (2004). Conserved residues in the Plasmodium vivax Duffy-binding protein ligand domain are critical for erythrocyte receptor recognition.. Proc Natl Acad Sci U S A.

[13] Singh SK, Singh AP, Pandey S, Yazdani SS, Chitnis CE (2003). Definition of structural elements in Plasmodium vivax and P. knowlesi Duffy-binding domains necessary for erythrocyte invasion.. Biochem J.

[14] Cole-Tobian J, King CL (2003). Diversity and natural selection in Plasmodium vivax Duffy binding protein gene.. Mol Biochem Parasitol.

[15] Kho WG, Chung JY, Sim EJ, Kim DW, Chung WC (2001). Analysis of polymorphic regions of Plasmodium vivax Duffy binding protein of Korean isolates.. Korean J Parasitol.

[16] Tsuboi T, Kappe SH, al-Yaman F, Prickett MD, Alpers M (1994). Natural variation within the principal adhesion domain of the Plasmodium vivax duffy binding protein.. Infect Immun.

[17] Bai T, Becker M, Gupta A, Strike P, Murphy VJ (2005). Structure of AMA1 from Plasmodium falciparum reveals a clustering of polymorphisms that surround a conserved hydrophobic pocket.. Proc Natl Acad Sci U S A.

[18] Coley AM, Parisi K, Masciantonio R, Hoeck J, Casey JL (2006). The most polymorphic residue on Plasmodium falciparum apical membrane antigen 1 determines binding of an invasion-inhibitory antibody.. Infect Immun.

[19] Crewther PE, Matthew ML, Flegg RH, Anders RF (1996). Protective immune responses to apical membrane antigen 1 of Plasmodium chabaudi involve recognition of strain-specific epitopes.. Infect Immun.

[20] Healer J, Murphy V, Hodder AN, Masciantonio R, Gemmill AW (2004). Allelic polymorphisms in apical membrane antigen-1 are responsible for evasion of antibody-mediated inhibition in Plasmodium falciparum.. Mol Microbiol.

[21] Pizarro JC, Vulliez-Le Normand B, Chesne-Seck ML, Collins CR, Withers-Martinez C (2005). Crystal structure of the malaria vaccine candidate apical membrane antigen 1.. Science.

[22] Smith DJ, Lapedes AS, de Jong JC, Bestebroer TM, Rimmelzwaan GF (2004). Mapping the antigenic and genetic evolution of influenza virus.. Science.

[23] VanBuskirk KM, Cole-Tobian JL, Baisor M, Sevova ES, Bockarie M (2004). Antigenic drift in the ligand domain of Plasmodium vivax duffy binding protein confers resistance to inhibitory antibodies.. J Infect Dis.

[24] Choe H, Moore MJ, Owens CM, Wright PL, Vasilieva N (2005). Sulphated tyrosines mediate association of chemokines and Plasmodium vivax Duffy binding protein with the Duffy antigen/receptor for chemokines (DARC).. Mol Microbiol.

[25] Singh SK, Hora R, Belrhali H, Chitnis CE, Sharma A (2006). Structural basis for Duffy recognition by the malaria parasite Duffy-binding-like domain.. Nature.

[26] Grimberg BT, Udomsangpetch R, Xainli J, McHenry A, Panichakul T (2007). Plasmodium vivax invasion of human erythrocytes inhibited by antibodies directed against the Duffy binding protein.. PLoS Med.

[27] Nakamura Y, Gojobori T, Ikemura T (2000). Codon usage tabulated from international DNA sequence databases: status for the year 2000.. Nucleic Acids Res.

[28] Betts MJ, Russell RB, Barnes MR, Gray IC (2003). Amino acid properties and consequences of substitutions.. Bioinformatics for Geneticists.

[29] Ampudia E, Patarroyo MA, Patarroyo ME, Murillo LA (1996). Genetic polymorphism of the Duffy receptor binding domain of Plasmodium vivax in Colombian wild isolates.. Mol Biochem Parasitol.

[30] Cole-Tobian JL, Cortes A, Baisor M, Kastens W, Xainli J (2002). Age-acquired immunity to a Plasmodium vivax invasion ligand, the duffy binding protein.. J Infect Dis.

[31] Xainli J, Adams JH, King CL (2000). The erythrocyte binding motif of plasmodium vivax duffy binding protein is highly polymorphic and functionally conserved in isolates from Papua New Guinea.. Mol Biochem Parasitol.

[32] Hans D, Pattnaik P, Bhattacharyya A, Shakri AR, Yazdani SS (2005). Mapping binding residues in the Plasmodium vivax domain that binds Duffy antigen during red cell invasion.. Mol Microbiol.

[33] Michon P, Fraser T, Adams JH (2000). Naturally acquired and vaccine-elicited antibodies block erythrocyte cytoadherence of the Plasmodium vivax Duffy binding protein.. Infect Immun.

[34] Michon P, Woolley I, Wood EM, Kastens W, Zimmerman PA (2001). Duffy-null promoter heterozygosity reduces DARC expression and abrogates adhesion of the P. vivax ligand required for blood-stage infection.. FEBS Lett.

[35] Arnold K, Bordoli L, Kopp J, Schwede T (2006). The SWISS-MODEL workspace: a web-based environment for protein structure homology modelling.. Bioinformatics.

[36] Schwede T, Kopp J, Guex N, Peitsch MC (2003). SWISS-MODEL: An automated protein homology-modeling server.. Nucleic Acids Res.

